# The Effectiveness of Positive Parenting Program (Triple-P) on General Health of Mothers of Pre-School Children with Intellectual Disability

**Published:** 2019-11

**Authors:** Mohammad ASHORI, Seyyedeh Somayyeh JALIL-ABKENAR

**Affiliations:** 1. Department of Psychology and Education of Children with Special Needs, Faculty of Education and Psychology, University of Isfahan, Isfahan, Iran; 2. Department of Psychology and Exceptional Children Education, School of Psychology and Educational Sciences, University of Tehran, Tehran, Iran

## Dear Editor-in-Chief

The concept of intellectual disability (ID) represents a contemporary change from mental retardation, initially coined as an option to feeblemindedness. Since 2002, the shift to ID has clearly increased (1).The birth of a child with ID cause the family many problems since it influences the quantity and quality of parent relationships and family members’ interaction could harm the general health and family functions (2). General health is an important issue which can spark many debates (3). “Mental health is also a state of well-being that person identifies their abilities, can cope with ordinary life stress, work fruitful and productive, and play his role in the environment” (4). Several factors are effective on general health of parents and their relationships with children with ID that require to different instructional methods and programs, that Positive Parenting Program (Triple-P) intervention based on cultural values is one of these programs. Triple-P intervention is a parent instruction methods made according to familial behavior therapy based on social learning theory basics of Bandura (5). The effect of Triple-P confirmed in treatment of behavioral problems and parenting styles of parents ineffective (6). The present study was aimed to assess the effect of Triple-P intervention on general health of mothers of pre-school children with ID in September 2017 in Varamin Province, Iran.

The present research was a quasi-experimental study by pretest, posttest design with a control group. Informed consent obtained from participants and present study approved by the Exceptional Education Organization of Tehran Province. Overall, 28 mothers of pre-school children with ID aged 5 to 6 years participated in this study selected by convenient sampling method from exceptional schools of Varamin Province, Iran. The subjects were divided into two groups; each group consisted of 14 individuals. The experimental group received parent training program in 10 sessions lasting for 70 min, while the control group did not. The instrument of present research was General Health Questionnaire (GHQ). Representative indexes of GHQ and subscales in pre-test and post-test of the experimental and control groups (Table 1).

**Table 1:** Descriptive indexes of experimental and control groups

***Variables***		***Time***	***Experimental group***		***Control group***	
			***Mean***	***SD***	***Mean***	***SD***
GHQ	Somatic symptoms	Pre-test	7.98	1.24	7.97	0.98
		Post-test	6.32	1.18	7.94	1.15
	Anxiety	Pre-test	9.93	1.37	10.02	1.29
		Post-test	7.45	1.20	9.99	1.01
	Social dysfunction	Pre-test	10.81	1.06	10.80	1.19
		Post-test	8.86	1.38	10.76	1.08
	Depression	Pre-test	9.90	1.11	9.84	1.24
		Post-test	7.24	1.07	9.84	1.17
	Total general health	Pre-test	38.62	2.99	38.63	2.85
		Post-test	29.87	2.86	38.53	2.93

Multivariate analysis of covariance (MANCOVA) was used because of presence of one independent variable and five dependent variables and moderate pre-test effect (7). After checking and confirmation of the normality of research variables, Box's test approved equality of variance-covariance. Moreover, approved assumption of variance equality by using Leven’s test. Therefore, MANCOVA test can be used. GHQ calculated via Wilk’s Lambda test, experimental and control group had significant difference, at least in one variable with eta square (η^2^), 84% (F=241.03, *P*=0.001). In order to determine this difference MANCOVA test was used, which results are showed in Table 2.

**Table 2:** Results of MANCOVA in of GHQ

***Dependent variable***		***SS***	***df***	***MS ***	***F***	**P**	***η^2^***
GHQ	Somatic symptoms	24.21	1	24.21	11.27	0.001	0.52
	Anxiety	40.67	1	40.67	20.65	0.001	0.57
	Social dysfunction	19.35	1	19.35	14.19	0.001	0.55
	Depression	21.18	1	21.18	13.44	0.001	0.53
	Total general health	61.59	1	61.59	19.60	0.001	0.56

In this analysis, pre-test variables were moderated because of correlation with post-test. According to Table 2, group had significant effect on post-test scores (*P*<0.05). According to eta square (η2), can be explained that 52%, 57%, 55%, 53% and 56% of the variation in each one of the variables of somatic symptoms, anxiety, social dys-function, depression, and total general health of GHQ, respectively are for the effect of Triple-P intervention on general health of participants.

Present research opposed with limitations such as: limiting in sample size, quasi-experimental research method and, due to time limitation, opportunity for follow-up was not provided. Future studies would be done on other exceptional children with diverse range of age and the effects of probable factors would be investigated and opportunities for follow-up of results would be provided.
